# Dataset for the analysis of gendered research productivity affected by early COVID-19 pandemic

**DOI:** 10.1016/j.dib.2023.109200

**Published:** 2023-05-04

**Authors:** Eunrang Kwon, Jinhyuk Yun, Jeong-han Kang

**Affiliations:** a Department of Sociology, Yonsei University, 50, Yonsei-ro, Seodaemun-gu, Seoul, 03722, (South Korea); b School of AI Convergence, Soongsil University, 369, Sangdo-ro, Dongjak-gu, Seoul, 06978, (South Korea)

**Keywords:** COVID-19, Gender inequality, Research productivity, Career, Childcare, Microsoft academic graph

## Abstract

In many countries, COVID-19 has made it harder for women to study because they are expected to do more housework and care for children. This article encompasses different data sources that can be used to figure out how the early pandemic of COVID-19 affected the number of studies done by females, in comparison with males. This data is add-on metadata that can be used with raw Microsoft Academic Graph (MAG) from 2016 to 2020 of the Feb 6, 2021 dump. We retrieved open-source metadata from various sources, including LinkedIn, the Johns Hopkins Coronavirus Resource Center, and Google's COVID-19 Community Mobility Reports, and linked bibliographic information to characteristics of the author's environments. It consists of published journals and online preprints, including each author's gender and involvement in the publication, their position through time, the h-index of their institutes, and gender equality in the professional labor market at the country level. For each record of papers, the data also includes the information of the papers, e.g., title and field of study. By gathering this evidence, our data can support the fact diversity in science is more than just the number of active members of different groups. It should also examine minority participation in science. Our data may help scholars understand diversity in science and advance it. The article ``The effect of the COVID-19 pandemic on gendered research productivity and its correlates'' uses this data as the principal source (Kwon, Yun & Kang, 2021).

**Specifications Table**

**Table:** 

Subject	Social Science
Specific subject area	The data include bibliographic information and author characteristics across all fields to analyze the research productivity of female authors during COVID-19.
Type of data	Python Codes Data
How the data were acquired	All data are downloaded from sources (see Description of data source location for more).
Data format	Raw
Description of data collection	Bibliographic data is extracted and processed from Microsoft Academic Graph. It consists of two paper types: journal and repository. We also collected information about the author's characteristics at the country level from external sources. It covers a total of 5 years from 2016 to 2020, except when the year of the date is not accurate.
Data source location	Bibliographic metadata of academic papers were retrieved from the Microsoft Academic Graph (https://academic.microsoft.com/; https://aka.ms/msracad) on Azure Storage (https://azure.microsoft.com/) licensed under ODC-BY. The gender disambiguation data set is owned by Demografix ApS and can be accessed from their website by means of a subscription (https://genderize.io/). Additional socio-economic data sets are also available from the following sources: epjds-professional-gender-gaps (https://github.com/fverkroost/epjds-professional-gender-gaps) for gender equality; CSSE (https://github.com/CSSEGISandData/COVID-19) for COVID-19 statistics; COVID-19 Community Mobility Reports (https://www.google.com/covid19/mobility) for mobility.
Data accessibility	Repository name: Mendeley Data Data identification number: DOI:10.17632/s3wjsv8mrb.1 Direct URL to data: https://data.mendeley.com/datasets/s3wjsv8mrb/1
Related research article	Kwon, E., Yun, J., & Kang, J. H. (2023). The effect of the COVID-19 pandemic on gendered research productivity and its correlates. Journal of Informetrics, 101380.

## Value of the Data

• These data compiled from MAG and other sources can be used to examine the evolution of research productivity before and after the COVID-19 outbreak. Since the data include bibliographic information for the five years between 2016 and 2020 across all disciplines, it can be used to compare before and after the COVID-19 outbreak on a discipline-by-discipline basis.
• The data also combined the bibliographic information supplied by MAG with open data sources to generate the author's background variables, such as gender. Through this method, it is possible to examine the productivity of female researchers, which varies across time, disciplines, and author and country characteristics.
• Researchers interested in gender inequality and discrimination in the labor market, including academic fields in terms of paper productivity, can benefit from these data. They can also provide researchers with useful variables for determining the early impact of COVID-19 on academia and potential factors affecting the author's research productivity.
• We provide datasets and codes for calculating research output using a variant of Difference-in-Difference (DID). It can be utilized as an indicator of the annual change in research productivity.
• The bibliographic data identify each paper's authors and provide their country of origin based on the location of their affiliation. It is possible to conduct additional research by combining external metadata based on the author's country. Researchers can also use these data for field-by-field analysis, given that data provides information field by field.
• To improve gender equality in society cares for female education enrollment and labor participation at the early stage and, once those missions are accomplished, moves to advanced criteria such as proportions of female senior managers and female empowerment in politics. Increasing diversity is also an important theme in the science of science and also should expand to more nuanced and advanced criteria. Our research suggests that measuring diversity in science should include the role and status of minorities in scientific production beyond the sheer volume of their participation. Our data include those measurements such as each author's gender and role in the paper, their time-varying status as well as universities’ status based on h-index, and country-level gender equality in the professional labor market. We vision that our data can facilitate more nuanced research on diversity in science and contribute to improving diversity in science to the next level.

## 1 Objective

During the COVID-19 period, female researchers may have encountered more challenges than their male counterparts due to gendered housework and childcare responsibilities. Our data were collected to examine how the productivity of female researchers has changed since early COVID-19 outbreak. Depending on the author's background, the research output of female authors can vary. For this purpose, the dataset spans a total of five years, from 2016 to 2020, and author characteristics variables were separated into three levels: individual, organizational, and country levels, respectively. Variables at the individual level include academic age and author h-index. The h-index for the author's affiliation was computed at the organizational level. The severity of COVID-19, mobility during the pandemic, and gender equality are included at the country level. The data also include the female role of each paper, as researchers who cannot devote a great deal of time to papers due to COVID-19 may prefer less time-intensive roles, thus moving their role from the leading authors to the others.

## 2 Data Description

We present four datasets and codes for analyzing the early impact of the COVID-19 pandemic on gendered research productivity. The data were filtered using bibliographic information from Microsoft Academic Graph (MAG) [1] and combined with open data sources. ``01_MAG_setting_dataset.ipynb'' is the file containing the code that generates the dataset. We provide offline journals and repositories as data sources. The code for gendered research productivity in offline journals is ``02_MAG_analysis_offline.ipynb,'' while the code for online repositories is ``03_MAG_analysis_online.ipynb.''

The ``Offline Dataset'' subdirectory contains the text files ``offline_DIB.txt'', ``author_role_DIB.txt'', ``effect_var_DIB.txt'', and ``author_order_DIB.txt''. The ``paperid'' and ``authorid'' columns, which offer unique identifiers for publications and authors, respectively, can be used to combine these datasets. 1) ``offline_DIB.txt'' is a dataset of journal papers. This dataset includes the (offline) publication date of the paper, the gender of the author by author's role, the number of authors, the paper's field, and the author's country of affiliation. 2) ``author_role_DIB.txt'' is an author-level dataset that contains information about each paper's authors. It provides the author's unique id, gender, and contribution to the paper. 3) ``effect_var_DIB.txt'' is an author-level dataset including author-related characteristics. It provides information on author h-index, affiliation h-index, and academic age. It also includes characteristics at the country level, such as gender equality, the severity of COVID-19, and migration throughout the epidemic. These characteristics were coupled with author information based on the author's country of association. 4) ``author_order_DIB.txt'' is a paper-level file. It contains the lists of papers that authors sorted in alphabetical order of the last name. The subfolder ``Online Dataset'' contains ``online_DIB.txt'', ``author_role_online_DIB.txt.'', and ``author_order_online_DIB.txt'' It is bibliographical information for the online repository, which is a preprint, and the data structure is identical to that of the offline dataset subfolder.

The definitions of all variables in the bibliographic data are as follows:

For the variables in the ‘Offline Dataset’ sub-folder:

(1) For the variables in the ‘offline_DIB.txt’:
  1. paperid: the unique id of a paper.
  2. jourid: the unique id of a journal.
  3. date: the date of offline publication.
  4. year: the year of offline publication.
  5. month1: the month of offline publication.
  6. female_have: a binary indicator of whether one of the paper's authors is female. If all the authors are male, the value is 0, and if at least one of the paper's authors is female, the value is 1.
  7. female_first: a binary indicator of whether the first author is female. If the first author is male, the value is 0, and if the first author is female, the value is 1.
  8. female_last: a binary indicator of whether the last author is female. If the last author is male, the value is 0, and if the last author is female, the value is 1.
  9. female_other: a binary indicator of whether the other authors except either the first or the last author is female. If these other authors are all male, the value is 0, and otherwise, the value is 1.
  10. author_n: the number of authors in the paper.
  11. field_id1: the academic field of a paper (A 'discipline.txt' file provides full descriptions of the fields).
  12. country1: the country of the first author's affiliation.
(2) For the variables in the ‘author_role_DIB.txt’:
  1. paperid: the unique id of a paper.
  2. authorid: the unique id of an author.
  3. year: the year of an offline publication.
  4. field_id1: the field of a paper.
  5. gender: the gender of an author.
  6. author_role: the role of an author in the paper. We divided the roles of authors into first author, last author, and other authors. For a paper with a single author, we attribute the first author designation to the author.
(3) For the variable in the ‘effect_var_DIB.txt’:
  1. authorid: the unique id of an author.
  2. affiliation: the unique id of an author's affiliation.
  3. aff_hidx: the h-index of an author's affiliation.
  4. aut_hidx: the h-index of an author.
  5. aca_age: the years to the present from the year of the author's first published paper.
  6. iso_code_3: country codes of 3digit.
  7. iso_code: country codes of 2digit.
  8. total_cases_per_million: the number of cases per million people from COVID-19 by 2020 by country.
  9. total_deaths_per_million: the number of deaths per million people from COVID-19 by 2020 by country.
  10. Workplace: the mobility in the workplace during COVID-19 by country.
  11. Residential: the mobility in the residential area during COVID-19 by country.
  12. ggis: the degree of gender equality within occupations by country.
(4) For the variables in the ‘author_order_DIB.txt’:
  1. paperid: the unique id of a paper that authors sorted in alphabetical order of the last name.
  2. author_n: the number of authors in the paper. This data does not include sign author papers.

For the variables in the ‘Online Dataset’ sub-folder:

(1) For the variable in the ‘online_DIB.txt’:
  1. paperid: the unique id of a paper.
  2. jourid: the unique id of a journal.
  3. onlinedate: the date of online publication.
  4. year: the year of online publication.
  5. month2: the month of online publication.
  6. female_have: a binary indicator of whether one of the paper's authors is female. If all the authors are male, the value is 0, and if at least one of the paper's authors is female, the value is 1.
  7. female_first: a binary indicator of whether the first author is female. If the first author is male, the value is 0, and if the first author is female, the value is 1.
  8. female_last: a binary indicator of whether the last author is female. If the last author is male, the value is 0, and if the last author is female, the value is 1.
  9. female_other: a binary indicator of whether the other authors except either the first or the last author is female. If these other authors are all male, the value is 0, and otherwise, the value is 1.
  10. author_n: the number of authors in the paper.
  11. field_id1: the academic field of a paper (A 'discipline.txt' file provides full descriptions of the fields).
  12. country1: the country of the first author's affiliation.
(2) For the variable in the ‘author_role_online_DIB.txt’:
  1. paperid: the unique id of a paper.
  2. authorid: the unique id of an author.
  3. year: the year of online publication.
  4. field_id1: the field of a paper.
  5. gender: the gender of an author.
  6. author_role: the role of an author in the paper. We divided the roles of authors into first author, last author, and other authors. For a paper with a single author, we attribute the first author designation to the author.
(3) For the variables in the ‘author_order_online_DIB.txt’:
  1. paperid: the unique id of a paper that authors sorted in alphabetical order of the last name.
  2. author_n: the number of authors in the paper. This data does not include single author papers.

## 3 Experimental Design, Materials and Methods

The data is generated through the post-process of bibliographic data from Microsoft Academic Graph (MAG) [1]. This data includes bibliographic information for 5 years from 2016 to 2020 to examine the early effect of COVID-19. MAG has ceased collecting data since December 6, 2021. Although OpenAlex continues collecting bibliographic data in succession, it is challenging to combine it with MAG because OpenAlex collects metadata using a different methodology [2]. Therefore, this data can be used by analyzing the early impact until 2020 and it is necessary to consider a new dataset for the middle or late effect of the COVID-19 outbreak.

MAG's ``Papers'' and ``PaperFieldsOfStudy'' tables were utilized to extract data at the paper level. The ``Papers'' table contains information such as the type of paper, the journal's unique id, and the date of publication. We assigned additional metadata of the papers using MAG bibliographic data and other sources as the following procedure. We first construct two types of datasets for analyzing published offline papers and preprints. To assign the publication date, we use the ``Date'' columns when the type of paper is ``journal'' for the offline (journals) dataset, whereas we use the ``OnlineDate'' when the type of paper is ``repository'' for the preprint (online) dataset. The papers' fields of study were also extracted from the ``PaperFieldsOfStudy'' table. MAG categorized fields into a total of 19 Level 0 fields, with the confidence score indicating the extent to which the paper corresponds to a certain field. Additionally, we divide them into five categories (Humanities, Social Science, Natural Science, Applied Science, and Medicine). For more detailed descriptions of the tables and columns, MAG provides Microsoft academic graph schema (https://learn.microsoft.com/en-us/academic services/graph/reference-data-schema).

The paper may have multiple authors. We thus use the ``Authors'', ``PaperAuthorAffiliations'', and ``Affiliations'' tables to add the authors' information for the papers. We count the number of authors in each publication and exclude those with over 50 authors. Since the authors' genders were not assigned by MAG, we use Genderize (https://genderize.io/) to assign the gender from the ``NormalizedName'' column of the ``Authors'' table. Generize.io is the API for classifying the gender of a person based on their name, social network user profiles, and census data. As a result of comparing the five gender prediction methods (SSA, IPUMS, Sexmachine, Genderize, and Face++) [3,4], we selected Genderize, which shows the best performance, the to classify gender of authors. The accuracy of Genderize is the second highest and Female and male precision shows the highest score compared five gender prediction methods. Genderize collected a total of 113,393,060 names, more than any other gender predictor, including about 80 percent English-speaking names and about 20 percent Asian names.

After separating the author's name with spaces, the gender is inferred from the first token as the first name. We categorized the author's gender using the Python package named genderize (https://pypi.org/project/Genderize/), and we treat it as a missing value when the name cannot be predicted due to insufficient data. The ``AuthorSequenceNumber'' column in the ``PaperAuthorAffiliations'' table can be used to assign the first and last authorship to authors. To distinguish the authorship of the authors within the paper, we assume he/she is the first author when the sequence number is one and the last author when the sequence number is the last. If authors are not classified as the first or last author, they are classified as other authors. The ``Affiliations'' table shows the author's affiliation and the country where the author's institution is located, recorded in the ``Iso3166Code'' column.

Academic age is calculated as the number of years between 2020 and the year the author's first article appeared in MAG. We retrieve the first published paper by each author from the ``Papers'' section. The author and affiliation h-indexes reveal the author's background, which may influence academic stability during the pandemic. The h-index is a citation-based metric defined as the greatest number of h papers published by a given author or affiliation that have been cited at least h times each [5]. To calculate the h-index for both authors and affiliations, we extracted all citations from the ``PaperReferences'' table and computed the number of citations received by each paper.

We use external open datasets to add variables at the national level, including gender equality, the severity of COVID-19, and mobility during COVID-19 in each country. The LinkedIn Gender Gap Index (GGI) is a national gender equality index for professional occupations that calculates the gender gap in their respective fields [6]. Because it is an aggregate indicator of the female-to-male ratio of LinkedIn Users, a higher value can be interpreted as a sign of a more gender-balanced country in terms of occupations. The Johns Hopkins Coronavirus Resource Center provides the total number of COVID-19 infections and its morality [7]. We compute cumulative country statistics until December 13, 2020. We also utilized Google's Covid-19 Community Mobility Reports [8] This data examines the differences in mobility that may change as a result of pandemic lockdowns. Among six categories of mobility index in the dataset, we select two mobility indexes (Workplaces and Residential mobility). Each mobility was computed as an average between the report's initial date and December 31, 2020. For instance, if residential mobility has a lower score, it indicates less movement at home than pre-corona mobility.

## Ethics Statements

Our work does not involve studies with animals and humans.

## CRediT authorship contribution statement

**Eunrang Kwon:** Data curation, Formal analysis, Conceptualization, Methodology, Writing – original draft, Writing – review & editing. **Jinhyuk Yun:** Conceptualization, Methodology, Writing – original draft, Writing – review & editing. **Jeong-han Kang:** Conceptualization, Methodology, Writing – original draft, Writing – review & editing.

## Declaration of Competing Interest

The authors declare that they have no known competing financial interests or personal relationships that could have appeared to influence the work reported in this paper.

## Data Availability

Dataset for the analysis of gendered research productivity affected by COVID-19 PandemicDataset for the analysis of gendered research productivity affected by COVID-19 Pandemic (Original data) (Mendeley Data).
